# Supplementary material for: Clinical Implication of Circulating Tumor Cells Expressing Epithelial Mesenchymal Transition (EMT) and Cancer Stem Cell (CSC) Markers and Their Perspective in HCC: A Systematic Review
Source: Cancers (Basel). 2022 Jul 11;14(14):3373. doi: 10.3390/cancers14143373 (PMC9322939; doi:10.3390/cancers14143373)
Supplement: Supplementary file 1 [file cancers-14-03373-s001.zip › cancers-1760181-supplementary.pdf]

cancers

MDPI

Supplementary Materials

Clinical Implication of Circulating Tumour Cells Expressing Epithelial Mesenchymal Transition (EMT) and Cancer Stem Cell (CSC) Markers and Their Perspective in HCC: A Systematic Review

Table S1. Search strategy in different database based on EMT and CSC markers expression in CTCs.

Database	Queries	Number of Papers
PubMed	((((((((("Liver cancer")) OR (("Hepatocellular carcinoma")) OR ((HCC)))) AND (((((("Circulating tumor cell")) OR (("Circulating tumour cell")) OR ((CTC)))) AND (("Epithelial mesenchymal transition")))) AND ((((Metastasis)) OR ((Recurrence)) OR ((Prognosis)))))) OR ((((liver cancer[MeSH Terms])) AND ((((Circulating tumor cell[MeSH Terms])) AND ((Epithelial mesenchymal transition[MeSH Terms])))) AND ((((Metastasis[MeSH Terms])) OR ((Recurrence[MeSH Terms])) OR ((Prognosis[MeSH Terms]))))))))) OR (((((((("Liver cancer")) OR (("Hepatocellular carcinoma")) OR ((HCC)))) AND (((((("Circulating tumor cell")) OR (("Circulating tumour cell")) OR ((CTC)))) AND (((("Cancer stem cells")) OR (("Circulating cancer stem cells")) OR (("Circulating tumor stem cells")))))) AND ((((Metastasis)) OR ((Recurrence)) OR ((Prognosis)))))) OR ((((liver cancer[MeSH Terms])) AND ((((Circulating tumor cell[MeSH Terms])) AND ((Cancer stem cells[MeSH Terms])))) AND ((((Metastasis[MeSH Terms])) OR ((Recurrence[MeSH Terms])) OR ((Prognosis[MeSH Terms]))))))))))	60
Embase	(('liver cancer' OR 'hepatocellular carcinoma' OR hcc) AND (('circulating tumor cell' OR 'circulating tumour cell' OR ctc) AND 'epithelial mesenchymal transition') AND (metastasis OR recurrence OR prognosis)) OR (('liver cancer' OR 'hepatocellular carcinoma' OR hcc) AND (('circulating tumor cell' OR 'circulating tumour cell' OR ctc) AND ('cancer stem cells' OR 'circulating cancer stem cells':af OR 'circulating tumor stem cells')) AND (metastasis OR recurrence OR prognosis))	82
Scopus	(TITLE-ABS-KEY ((((*"liver cancer"*) OR (*"hepatocellular carcinoma"*) OR (*hcc*)) AND ((*"epithelial mesenchymal transition"*) AND ((*"circulating tumor cell"*) OR (*ctc*))) AND ((*metastasis*) OR (*recurrence*) OR (*prognosis*)))) OR TITLE-ABS-KEY (((((*"liver cancer"*) OR (*"hepatocellular carcinoma"*) OR (*hcc*)) AND (((*"circulating tumor stem cells"*) OR (*"cancer stem cells"*) OR (*"circulating cancer stem"*)) AND (*"circulating tumor cell"*) OR (*ctc*)) AND ((*metastasis*) OR (*recurrence*) OR (*prognosis*))))))	72

Table S2. Risk of bias assessment (Newcastle–Ottawa Quality Assessment Scale criteria).

Study	Selection (0-4)				Comparability (0-2)	Outcome (0-3)			Total Score
	REC	SNEC	AE	DO		AO	FU	AFU	
Fan, 2011	0	0	1	1	1	1	1	0	5
Liu, 2013	0	0	0	1	2	0	0	1	4
Schulze, 2013	1	0	1	1	1	1	1	0	6
Court, 2018	1	0	1	1	2	1	1	0	7
Guo, 2018	1	0	1	1	1	1	1	0	6
Ou, 2018	0	0	1	1	2	1	1	0	6
Qi, 2018	0	1	1	1	1	1	1	1	7
Wang, 2018	0	0	1	1	1	1	1	1	6
Chen, 2019	0	0	1	1	2	1	1	1	7
Bai, 2020	0	0	1	1	2	1	1	0	6
Qi, 2020	1	0	1	1	2	1	1	1	8
Lei, 2021	0	1	1	1	2	0	1	1	7
Xie, 2021	0	0	1	1	1	1	1	1	6

REC, Representativeness of Exposed Cohort; SNEC, Selection of the Non-Exposed Cohort; AE, Ascertainment of Exposure; DO, Demonstration that Outcome of interest was not present at start of study; AO, Assessment of Outcome; FU, Follow-Up long enough for outcome to occur (≥6 months); AFU, Adequacy of Follow-Up.

Table S3. GRADE evidence profiles for studies in the meta-analysis.

Certainty Assessment							No. of Patients		Effect		
**No. of Studies**	**Study Design**	**Risk of Bias**	**Inconsistency**	**Indirectness**	**Imprecision**	**Other Considerations**	**CTCs Subtype Positive**	**CTCs Subtype Negative**	**Relative (95% CI)**	**Absolute (95% CI)**	**Certainty**
**Pre-operative mesenchymal CTCs (assessed with: HR)**											
2	observational studies	serious [a,b]	not serious	not serious	not serious	none			**HR 1.02** (1.01-1.03)	**N/A** [c]	⊕◯◯◯ Very low [a]
**Pre-operative epithelial CTCs (assessed with: HR)**											
2	observational studies	serious [a,b]	not serious	not serious	not serious	none			**HR 1.03** (0.88-1.20)	**N/A** [c]	⊕◯◯◯ Very low [a]
**Post-operative mesenchymal CTCs (assessed with: HR)**											
2	observational studies	serious [a,b]	not serious	not serious	not serious	strong association	33/62 (53.2%)	10/56 (17.9%)	**HR 4.56** (2.19–9.48)	**414 more per 1000** (from 171 more to 667 more)	⊕⊕◯◯ Low [a]

CI, confidence interval. HR, hazard Ratio. N/A, not applicate. [a] Most information is from studies at low or unclear risk of bias. [b] There is a limited number of studies for analysis. [c] The number of events were not reported.
